# Scaling preferences using probabilistic choice models: is there a ratio-scale representation of subjective liking?

**DOI:** 10.1007/s00426-022-01775-8

**Published:** 2022-12-03

**Authors:** Florian Kattner, Anne Gast

**Affiliations:** 1 Health and Medical University, Olympischer Weg 1, 14471 Potsdam, Germany; 2 University of Cologne, Cologne, Germany

## Abstract

In two online experiments, we tested whether preference judgments can be used to derive a valid ratio-scale representation of subjective liking across different stimulus sets. Therefore, participants were asked to indicate their preferences for all possible pairwise comparisons of 20 paintings (Experiment 1) and 20 faces (Experiment 2). Probabilistic choice models were fit to the resulting preference probabilities (requiring different degrees of stochastic transitivity), demonstrating that a ratio-scale representation of the liking of both paintings and faces can be derived consistently from the preference judgments. While the preference judgments of paintings were consistent with the highly restrictive Bradley–Terry–Luce model (Bradley and Terry, Biometrika 39:324–345, 1952; Luce, 1959), the liking of faces could be represented on a ratio scale only when accounting for face gender as an additional aspect in an elimination-by-aspects model. These ratio-scaled liking scores were then related to direct evaluative ratings of the same stimuli on a 21-point Likert scale, given both before and after the pairwise comparisons. It was found in both studies that evaluative ratings can be described accurately as a logarithmic function of the indirectly derived liking scores for both types of stimuli. The results indicate that participants are able (a) to consistently judge preferences across two heterogeneous stimulus sets, and (b) to validly report their liking in direct evaluative ratings, although the numeric labels derived from direct evaluative ratings cannot be interpreted at face value for ratio-scaled liking scores.

## Introduction

The use of direct evaluative ratings to measure subjective liking or disliking^1^ is ubiquitous in many areas of fundamental and applied research such as educational psychology (e.g., Greenwald & Gillmore, 1997), aesthetic judgments (e.g., Hekkert & van Wieringen, 1990) medicine (e.g., pain assessment scales; Gordon, 2015), and marketing research (e.g., Raines, 2003; Wildt & Mazis, 1978). It is particularly prevalent in social cognition research, for instance for measuring the changes in liking resulting from mere exposure (Zajonc, 1968) or evaluative conditioning (De Houwer et al., 2001). In social psychology, evaluative ratings are often used to study attitudes (Olson & Zanna, 1993), a central concept in social psychology (Allport, 1935).

In a typical ‘evaluative rating’ procedure, the participant is asked to report the degree of subjective liking or disliking of a given stimulus on a predetermined scale (e.g., a numerical rating scale, a visual analog scale, a feeling thermometer, or a scale with verbal labels such as ‘not at all’ and ‘very much’). Another approach to assess the liking of a stimulus is to ask the participant to make a comparative judgment with regard to two stimuli to indicate which of the two stimuli is liked more (evaluated more positively), and we refer to this measure as ‘preference judgments’.

Both evaluative ratings and preference judgments are forms of evaluative responses. Although there are debates about the relationship between an evaluative response and the underlying mental construct (e.g., Breckler, 1984; De Houwer et al., 2013, 2021; Krosnick et al., 2005; Kruglanski & Stroebe, 2005), most researchers assume that the evaluative response can be distinguished from a mental representation, which is sometimes referred to as the attitude (Eagly & Chaiken, 1993; Krosnick et al., 2005; Zanna & Rempel, 2008). Others see attitudes as complex entities, consisting of behavioral, cognitive, and affective components (Breckler, 1984; Rosenberg et al., 1960). In any case, the question arises how a presumably multidimensional construct such as an attitude or a subjective liking is translated into an evaluative rating (e.g., choosing a point on a one-dimensional scale) or a preference judgment.

From a psychophysical perspective, evaluative ratings are an example of direct scaling (Stevens, 1971), which requires participants to introspectively judge the intensity of a subjective liking of a stimulus and to be able to verbally report this intensity on a particular scale (e.g., ‘7’ on a scale from ‘1’ to ‘9’, or the 73% point on a visual analogue scale). Hence, it is implicitly assumed that (a) the underlying subjective liking is represented validly at a certain scale level and (b) the participants are able to verbally report their liking on a numerical rating scale (i.e., the chosen value on the scale can be interpreted as the intensity of liking). Unfortunately, it is not well understood whether participants’ direct evaluative ratings can be taken as a valid scale of their liking, and what level of measurement applies to this scale for any given set of stimuli (e.g., an ordinal or a ratio scale; Stevens, 1946). In other words, the validity of evaluative ratings as a method of direct scaling is questionable.

It is possible to address these questions empirically using scaling models that are based on axiomatic measurement theory, which specify the exact conditions that are necessary for direct measurement on a particular scale level (e.g., Iverson & Luce, 1998; Narens & Luce, 1986). The main advantages of such models are that (a) the mathematical pre-conditions of scaling on a particular level (e.g., on a ratio scale) can be tested empirically, and (b) the data collection is separated from the derivation of a scale (in contrast to direct scaling, which requires participants to respond with a numerical value on the scale itself, as in evaluative ratings). One such model is the Bradley–Terry–Luce (BTL) model (Bradley & Terry, 1952; Luce, 1959) allowing the derivation of a ratio scale of subjective liking from the preference judgments in a full set of (consistent) pairwise comparisons. The BTL model is a highly restrictive probabilistic choice model based on Luce’s choice axiom (Luce, 1959), assuming that the choice between multiple alternatives (i.e., preference judgments) is probabilistic and can be predicted as a function of the respective weights (*u*) of the alternatives (e.g., the intensity of liking of a stimulus). The empirically observed preference probabilities (${p}_{ab}$, indicating the probability of preferring stimulus *a* over stimulus *b* in a full pairwise comparison) can be related to the values on a ratio scale, representing the weights (or liking) *u* of the two stimuli (see Eq. 1).

1 $$p_{ab} = \frac{{u\left( a \right)}}{{u\left( a \right) + u\left( b \right)}} .$$

However, the BTL model makes very strong assumptions on the structure of the data, and a violation of these assumptions precludes fitting the model to the data. In particular, the model requires that the choice between two stimuli must be independent of the context provided by the entire stimulus set (i.e., context independence or independence of irrelevant alternatives). For example, the preference for clementine over grapefruit must be independent of the entire set of stimuli presented to the participant (e.g., whether the set consists of only citrus fruits or includes other food products such as cheesecake as well). Obviously, this assumption can fail especially in case of a multidimensional stimulus space or when there are similarities within certain subgroups of the stimuli (e.g., Carroll & Soete, 1991; Choisel & Wickelmaier, 2007; Debreu, 1960; Rumelhart & Greeno, 1971). The context independence property requires the observed preference judgments to be highly consistent, which can be tested empirically for any given set of data (i.e., full pairwise comparisons) in terms of violations of the transitivity axiom. Transitivity of preference judgments requires that whenever stimulus *a* is preferred over stimulus *b*, and this stimulus *b* is preferred over a third stimulus *c*, then *a* should be preferred over *c* as well. Different levels of stochastic transitivity can be distinguished with regard to the preference probabilities (see Eq. 2), and the BTL model requires strong stochastic transitivity (SST) to hold. According to the SST axiom, the probability to prefer stimulus *a* over stimulus *c* (${p}_{ac}$>0.5) must be larger than both the two probabilities to prefer *a* over *b* (${p}_{ab}$>0.5) and *b* over *c* (${p}_{bc}$>0.5). Importantly, if the restrictive BTL model cannot be fitted to the data due to systematic violations of SST (i.e., when the number of transitivity violation exceeds the number that would be expected by chance alone), then it may be possible to still derive a less restrictive probabilistic choice model, such as the generalized elimination-by-aspects (EBA) model (Tversky, 1972; Tversky & Sattath, 1979). The EBA model only requires moderate stochastic transitivity (MST), so that it is sufficient when the probability ${p}_{ac}$ is larger than any of the two probabilities ${p}_{ab}$ or ${p}_{bc}$. Finally, weak stochastic transitivity (WST) requires that the probability to prefer *a* over *c* must be greater than 0.5 whenever the probabilities ${p}_{ab}$ and ${p}_{bc}$ are greater than 0.5. A systematic violation of WST indicates that participants may not be able to integrate multiple relevant stimulus dimensions (e.g., symmetry, colorfulness, familiarity) on a single scale (e.g., liking), which makes it impossible to even derive a meaningful rank order of the stimulus likings (Choisel & Wickelmaier, 2007).

2 $$p_{ac} > \left\{ {\begin{array}{*{20}l} {{\text{max}}\left( {p_{ab} ,p_{bc} } \right)}  {(SST)} \\ {{\text{min}}\left( {p_{ab} ,p_{bc} } \right)}  {(MST)} \\ {0.5} {(WST)} \\ \end{array} } \right.$$

According to the EBA model (compare Eq. 3), participants are supposed to judge several features of stimuli separately (i.e., the aspects $\alpha ,\beta , \dots$), and aspects are chosen by the participant based on their individual weights (*u*). A stimulus *a* will be preferred over stimulus *b*, whenever a crucial aspect ($\alpha$; e.g., symmetry) is present in *a*, but not in *b* ($\alpha \in a^{\prime}\backslash b^{\prime}$)—or, in other words, stimuli not containing the crucial aspect will be eliminated successively. Technically, the BTL model is a special case of EBA with only one aspect per stimulus (i.e., a unidimensional stimulus set). However, in contrast to the BTL model, the EBA model does not require context independence.

3 $$p_{ab} = \frac{{\sum _{\alpha \in a^{\prime}\backslash b^{\prime}} u\left( \alpha \right)}}{{\sum _{\alpha \in a^{\prime}\backslash b^{\prime}} u\left( \alpha \right) + \sum _{\alpha \in a^{\prime}\backslash b^{\prime}} u\left( \beta \right)}} .$$

There have been several successful applications of the BTL model for the measurement of various subjective dimensions including pain (Matthews & Morris, 1995), taste and food quality (Lukas, 1991; Oberfeld et al., 2009), unpleasantness of sounds (Ellermeier et al., 2004; Zimmer et al., 2004), facial attractiveness (Bäuml, 1994; Kissler & Bäuml, 2000), as well as for university rankings (Dittrich et al., 1998) or preferences for insurance packages (McGuire & Davison, 1991).

The first purpose of this study was to test whether simple preference judgments can be described with a probabilistic choice model, allowing us to derive a ratio-scale representation of the underlying ‘liking’ weights of the stimuli. The preference judgments from a full pairwise comparison of all stimuli were then tested for weak, moderate, and strong stochastic transitivity. Based on the outcome of these consistency checks, a representation of the liking scores on a ratio scale was derived using either the BTL model or the generalized EBA model (note that both models can fail). The second objective of the present study was to test whether the commonly used direct evaluative ratings on a 21-point Likert scale are valid on a ratio scale, which would allow the interpretation of both ratios and differences between ratings on the scale at face value. Therefore, the direct evaluative ratings were described as a mathematical function of the ratio-scaled liking scores of the stimuli (the *u*-values) derived from the preference judgments using a probabilistic choice model. To demonstrate the generalizability, preference judgments and evaluative ratings were investigated for two qualitatively different sets of stimuli: abstract paintings and pictures of human faces (including males and females).

## Experiment 1

### Method

#### Participants

One hundred and forty-five participants (72 women, 73 men) were recruited via Prolific Academic (https://www.prolific.co/). Ages ranged between 18 and 73 years (*M* = 35.3; *Mdn* = 33; *SD* = 12.6). Participants were allowed to take part in the study only if they had normal or corrected-to-normal vision, English as their first language, and an approval rate of at least 95%. Five additional participants had been recruited, but they did not complete all tasks and their data was not included. All participants confirmed to participate voluntarily and agreed to an informed consent sheet by clicking on a checkbox before starting the experiment. The entire task took about 12 min on average and participants were compensated with £1.10 on Prolific.

#### Apparatus and stimuli

The evaluative rating and pairwise preference judgment tasks were programmed in JavaScript and conducted entirely online (using jsPsych; de Leeuw, 2015). A set of 20 pictures of paintings from four different artists (five pictures each from Jean Dubuffet, André Masson, Robert Motherwell, and John Wells) were chosen from the TATE Modern online art collections (https://www.tate.org.uk/art/artists/a-z) and used as the to-be-evaluated stimuli in Experiment 1. The particular pictures were selected to be of similar complexity, rather non-representational (i.e., abstract art) and from artists not very well known by the general population.

#### Procedure

The study started with the evaluative rating phase, asking participants to judge their liking of each of the 20 pictures on a 21-point Likert scale (which is typically used in studies on evaluative conditioning; e.g., Baeyens et al., 1990; Gast & Kattner, 2016; Hammerl & Grabitz, 2000) ranging from “totally dislike” (− 10) via “neutral” (0) to “totally like” (10). There was no time limit for clicking on the scale. The clicked position on the scale was highlighted for 250 ms, and the next rating started after a 500-ms blank screen interval. The order of the pictures was randomized for each participant.

After the first evaluative rating phase, participants were presented with all possible $\frac{n(n-1)}{2}=190$ combinations of two paintings in the preference judgments task. The paintings of each pair were shown simultaneously in the left and right half of the screen (the order was chosen randomly), and participants were asked to indicate which painting they liked better by clicking on the image. There was no time limit to the choice. The clicked picture was highlighted for 250 ms, and the next pair was presented after a 500-ms inter-trial interval with a blank screen. The order of the pairs was randomized for each participant.

After the preference judgments, participants were asked to provide another set of evaluative ratings of all stimuli using the same procedure as described above. At the end of the study, participants were asked to indicate whether they recognized any of the painters of the pictures by typing their names in a text box. Finally, participants should indicate how much they were interested in art on a 5-point scale from “not at all” to “very much”.

## Results

Only one participant recognized one of the painters correctly (“Motherwell”). Most participants reported to have recognized none of the pictures or artists, and there were only a few incorrect responses (e.g., “Klimt”, “Picasso”, “Klee”, or “Cezanne”), thus confirming that the artists were not well known in our sample. The average rating for the interest in art was 2.90 (*SD* = 1.16).

**Fig. 1:**
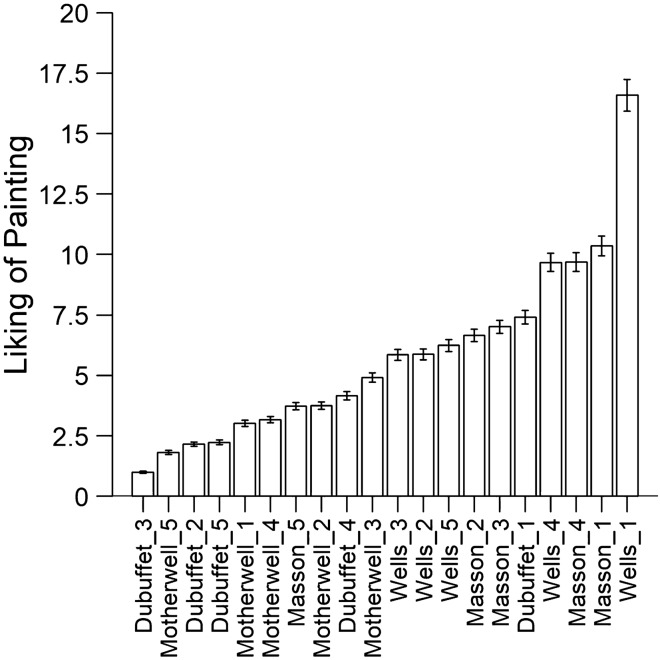
Estimated subjective liking of paintings on a ratio scale based on the BTL model. Error bars indicate standard errors of the model estimates

The pairwise preference judgments were first checked for consistency by testing the number of observed circular triads (when *a* > *b*, *b* > *c*, but *a* < *c*) against the number of circular triads that would be expected by chance alone (285 triads) using the{eba} package for R (Wickelmaier, 2020; Wickelmaier & Schmid, 2004). There was an average number of 39 circular triads out of a maximum of 330. In one participant, the number of circular triads did not differ significantly from chance performance (*T* = 279 circular triads; Kendall’s *zeta* = 0.15; *p* = 0.65), indicating highly inconsistent or random choice behavior, and the data of this participant were not included in the subsequent analyses. For the remaining participants, a cumulative preference matrix is shown in Table 1. This matrix contains the absolute frequencies of preferences of a painting in the row of the table over the painting in the respective column of the table. As a measure of consistency of the choice behavior across participants, stochastic transitivity was checked for all possible triads of stimuli (*a*, *b*, *c*) in the cumulative preference matrix. There were only two violations of WST (in 1140 tests), and an approximate likelihood ratio test (Iverson & Falmagne, 1985; Tversky, 1969) revealed that these violations were not significantly different from what would be expected by chance if transitivity held, *D* = 0.028; *p* = 0.87, thus indicating that an ordinal scale of the paintings can be derived from the preference choices. Further, there were also relatively few violations of the MST (12 triads; 1%) and SST criterion (206 triads; 18%). A BTL probabilistic choice model was then fitted to the preference probabilities to estimate the *u*-scale weights of liking of the 20 paintings using maximum-likelihood estimation (using the “OptiPt” function; Wickelmaier & Schmid, 2004). A likelihood ratio test was conducted to test the validity of the model, contrasting the likelihood of the BTL model with the likelihood of a restricted model assuming the preference probabilities to result from independent binomial distributions: $-2 \mathrm{ln}\left({L}_{\mathrm{BTL}}/{L}_{\mathrm{restricted}}\right)$. The *G*-test of goodness of fit revealed that the data did not deviate significantly from the BTL model, *G*^2^(171) = 164.4; *p* = 0.63, indicating that the BTL model provides an accurate description of the preferences. Therefore, it was possible to estimate the *u*-scaled values of the liking of all 20 paintings with a restrictive probabilistic choice model. An arbitrary value of 1 was assigned to the painting with the lowest *u*-scale value (“Dubuffet_3”), and all other values were scaled relative to this reference. The resulting ratio scale of the liking of painting is illustrated in Fig. 1. It can be seen that the least and most liked paintings are separated by a factor of 16.58, which suggests that (to our non-expert sample) the liking of painting “Wells_1” was more than 16 times stronger than the liking the painting “Dubuffet_3”.

**Table 1:** Cumulative preference matrix (*N* = 144) for 20 paintings from four different artists, depicting the absolute numbers of participants who preferred the painting in the row to the painting in the column

	Dubuffet 1–5					Masson 1–5				
Dubuffet 1	0	113	128	97	111	56	83	78	58	89
Dubuffet 2	31	0	105	49	66	29	35	31	29	47
Dubuffet 3	16	39	0	30	35	14	21	20	16	33
Dubuffet 4	47	95	114	0	98	42	63	41	42	77
Dubuffet 5	33	78	109	46	0	28	35	23	26	46
Masson 1	88	115	130	102	116	0	93	90	80	105
Masson 2	61	109	123	81	109	51	0	77	61	97
Masson 3	66	113	124	103	121	54	67	0	54	100
Masson 4	86	115	128	102	118	64	83	90	0	108
Masson 5	55	97	111	67	98	39	47	44	36	0
Motherwell 1	47	90	107	68	76	34	46	49	41	59
Motherwell 2	52	84	106	73	87	47	50	47	47	66
Motherwell 3	59	94	113	70	86	42	59	61	46	80
Motherwell 4	47	85	109	65	78	41	42	50	36	65
Motherwell 5	27	67	108	49	69	23	37	28	25	40
Wells 1	94	124	137	109	128	82	102	102	89	123
Wells 2	69	107	123	84	108	53	70	54	53	99
Wells 3	60	110	121	79	108	50	67	70	57	90
Wells 4	82	115	128	93	122	72	84	91	68	109
Wells 5	60	111	124	87	109	55	65	69	51	90

	Motherwell 1–5					Wells 1–5				
Dubuffet 1	97	92	85	97	117	50	75	84	62	84
Dubuffet 2	54	60	50	59	77	20	37	34	29	33
Dubuffet 3	37	38	31	35	36	7	21	23	16	20
Dubuffet 4	76	71	74	79	95	35	60	65	51	57
Dubuffet 5	68	57	58	66	75	16	36	36	22	35
Masson 1	110	97	102	103	121	62	91	94	72	89
Masson 2	98	94	85	102	107	42	74	77	60	79
Masson 3	95	97	83	94	116	42	90	74	53	75
Masson 4	103	97	98	108	119	55	91	87	76	93
Masson 5	85	78	64	79	104	21	45	54	35	54
Motherwell 1	0	61	39	66	100	22	56	48	31	38
Motherwell 2	83	0	56	84	96	33	60	53	41	49
Motherwell 3	105	88	0	93	108	37	66	67	52	66
Motherwell 4	78	60	51	0	99	23	52	49	34	45
Motherwell 5	44	48	36	45	0	17	37	25	27	26
Wells 1	122	111	107	121	127	0	110	119	94	114
Wells 2	88	84	78	92	107	34	0	74	59	71
Wells 3	96	91	77	95	119	25	70	0	56	64
Wells 4	113	103	92	110	117	50	85	88	0	98
Wells 5	106	95	78	99	118	30	73	80	46	0

**Fig. 2:**
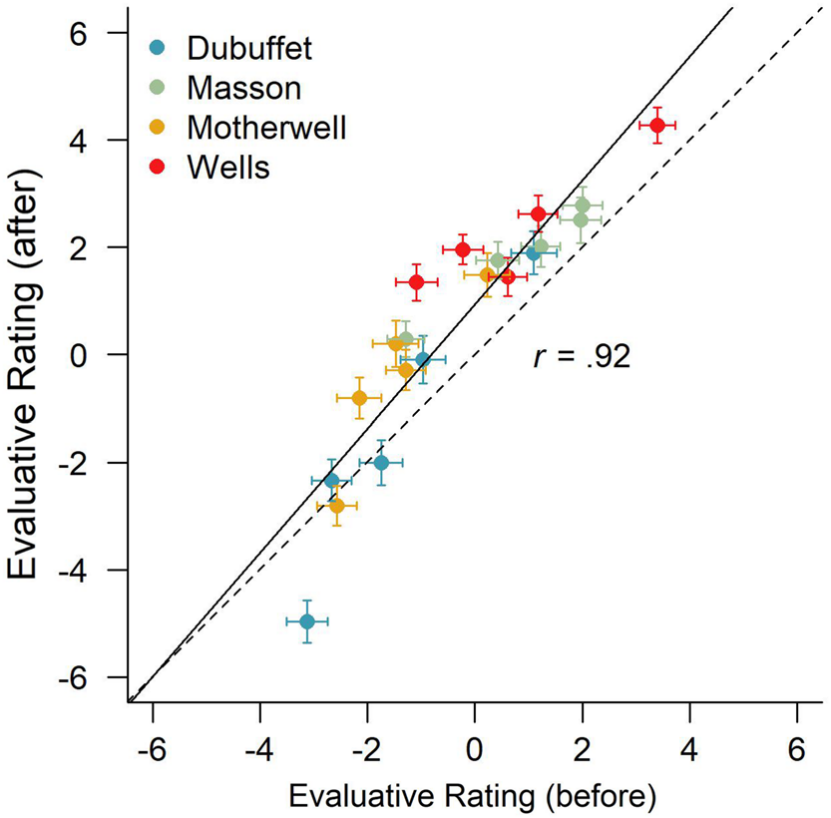
Correlation between the first evaluative ratings (before the pairwise preference judgments) and the second evaluative ratings (after the pairwise preference judgments) on a scale from − 10 to + 10 for the 20 paintings of four different artists presented in Experiment 1. *Error bars* indicate standard errors of the means. The *solid line* represents a linear regression between the two ratings, and the dashed line represents the diagonal

The average direct evaluative ratings in the first rating phase (before the pairwise comparisons) ranged between *M* = -3.13 (SD = 4.57; “Debuffet_3”) and *M* = 3.40 (SD = 4.01; “Wells_1”). There was a strong and significant correlation between the first and second evaluative ratings, *r* = 0.92; *t*(18) = 9.85; *p* < 0.001, indicating very good re-test reliability of the evaluative ratings (see Fig. 2). However, a 2 (time of measurement: before, after the preference judgments) × 4 (artist: Dubuffet, Masson, Motherwell, Wells) repeated-measures ANOVA revealed that the evaluative ratings also differed significantly between the first (*M* = − 0.32; SD = 2.60) and the second rating phase (*M* = 0.56; SD = 1.88), *F*(1,143) = 37.81; *p* < 0.001; *η*^2^_G_ = 0.02, demonstrating a general increase in liking with repeated exposure to the paintings (i.e., the majority of data points are above the diagonal in Fig. 2), possibly a mere exposure effect (Zajonc, 1968). In addition, there was a significant difference in evaluative ratings of paintings between the four artists (Dubuffet: *M* = − 1.49; SD = 3.14; Motherwell: *M* = − 0.95; SD = 3.37; Masson: *M* = 1.37; SD = 2.68; Wells: *M* = 1.55; SD = 2.48), *F*(3,429) = 62.34; *p* < 0.001; *η*^2^_G_ = 0.16. Finally, there was also a significant interaction between time of rating and artist, *F*(3,429) = 18.92; *p* < 0.001; *η*^2^_G_ = 0.01, indicating that the temporal change of evaluative ratings of paintings differed between the four artists (e.g., some artists fell closer to the diagonal than others in Fig. 2).

**Fig. 3:**
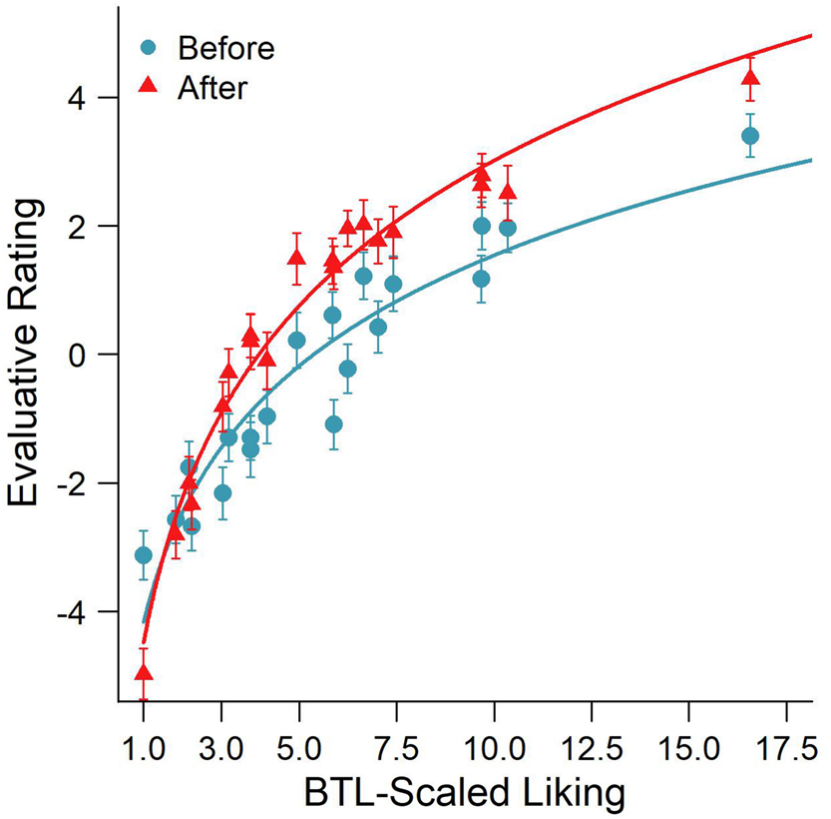
Evaluative ratings before and after the pairwise comparisons as a function of the BTL-scaled liking weights of the 20 paintings. The solid line is the best-fitting logarithmic regression predicting the first and second ratings as a function of the liking scores based on the BTL model

The relationship between the direct evaluative rating and the BTL-scaled *u*-values of the liking of the paintings (as derived from the pairwise preference judgments) is illustrated in Fig. 3. A non-linear regression using a least-squares method revealed that a two-parameter logarithmic function of liking scores *u* provided a good fit of the relationship between the evaluative ratings (*ER*) and the BTL-scaled liking weights (see Eq. 4; with $\alpha$ = 5.39; $\beta$ = 5.71 and $\alpha$ = 3.96; $\beta$ = 7.49 for the best fits of the evaluative ratings that were given before and after the pairwise comparisons, respectively).

4 $$\mathrm{ER}=\alpha \cdot \mathrm{log}\left(\frac{u}{\beta }\right).$$

Hence, a linear regression of the evaluative ratings as a function of the logarithm of the *u*-values of liking provided a very good fit of the data, accounting for almost 90% of the variance in the first evaluative ratings (*R*^2^ = 0.89) and even 97% of the variance in the second evaluative ratings (*R*^2^ = 0.97). This strong functional relationship indicates that a constant increment in evaluative ratings (e.g., + 2 points on the 21-point rating scale) corresponds to the multiplication of the subjective liking score with a certain factor (e.g., doubling the liking score on the ratio scale, as derived from preference judgments using a probabilistic choice model). In other words, the direct evaluative ratings cannot be interpreted on a ratio scale at face value (i.e., the numerical values on the rating scale are valid only on an ordinal scale), but they can be transformed to a ratio scale using an exponential function, allowing the interpretation of ratios between two liking values. Based on the present data, the ratio-scaled liking of paintings can be predicted as exponential functions of the first and second evaluative ratings (ER1 and ER2, provided in Eqs. 5 and 6), accounting for 72% and 74% of the variance.

5 $$u=4.31+0.47\cdot {e}^{ER1},$$

6 $$u=4.15+0.20\cdot {e}^{ER2}.$$

## Discussion

Experiment 1 demonstrated that the subjective liking of abstract paintings can be expressed consistently in a full set of preference judgments, enabling the estimation of ratio-scaled liking scores using a probabilistic choice model. Specifically, the fact that the data can be described with the highly restrictive BTL model proves that the subjective preference probabilities of the paintings from four different artists exhibit “context independence”, meaning that the preference judgments for any pair of paintings are based on the same evaluative aspects, both between and within different artists (i.e., the set of aspects that are relevant for the evaluation of paintings remains the same across contexts). In contrast, if different aspects had been considered depending on the context (e.g., different evaluative aspects in different artists), then the model would have failed statistically.

Since the subjective liking of paintings, as expressed in preference judgments, could be represented on a BTL ratio scale, we were able to relate this mathematically grounded ratio scale of liking scores to the direct evaluative ratings given by participants. In contrast to the indirect derivation of a BTL scale of liking scores (which requires only preference judgments), direct evaluative ratings are based on the untested assumption that participants are able to assign numerical values to the stimuli that provide a valid representation of the degree of subjective liking. In the present experiment, a typical evaluative rating procedure was used, asking participants to rate the liking of the paintings on a 21-point scale ranging from − 10 to + 10. Interestingly, these evaluative ratings—given both before and after the pairwise preference judgments with the same stimulus set—were found to be (a) highly consistent (i.e., high re-test reliability) and (b) strongly related to the BTL-scaled liking scores, thus demonstrating the general validity of direct evaluative ratings. Nevertheless, there was no simple linear relationship between the derived liking scores on the BTL scale and the directly reported evaluative ratings. The observation that evaluative ratings can be described accurately as a logarithmic function of the BTL-scaled liking scores suggests that directly reported values on the rating scale cannot be taken at face value and may not be valid on a ratio-scale level. Hence, differences and ratios between any two numerical values on the rating scale cannot be interpreted in terms of mathematical numbers (e.g., the difference between an evaluative rating of ‘3’ and ‘5’ may not be the same as the difference between a rating of ‘5’ and ‘7’, and an evaluative rating of ‘4’ does not mean that the stimulus is twice as pleasant as a stimulus with a rating of ‘2’). The strong relationship between direct evaluative ratings and a ratio-scale measure of subjective liking, however, suggests that an exponential transformation of the direct ratings may represent a valid ratio scale of the subjective likings.

## Experiment 2

To test the reliability and generalizability of the findings of Experiment 1, a second experiment was conducted using an entirely different set of stimuli (pictures of male and female human faces). Again, participants were asked to indicate the liking of the faces both in a full pairwise preference judgment task and through direct evaluative ratings. The procedures and data analysis strategies of Experiment 2 were pre-registered on OSF in January 2021, prior to the data collection: https://osf.io/h9d5k.

## Method

### Participants

A sample of 197 participants (155 women, 80 men, 1 queer, 1 nondisclosure) were recruited via Prolific Academic. The data of ten additional participants were incomplete and could not be included in the analyses. Participants were allowed to take part in the study only if the pre-screening confirmed normal or corrected-to-normal vision, English as their first language, an approval rate of at least 95%, and that they did not take part in Experiment 1. Ages ranged between 18 and 75 years (*M* = 33.7; Mdn = 33; SD = 12.3). The entire experiment took about 13 min, and all participants were compensated with £1.70 via Prolific (note that the duration was similar to Experiment 1, but the payment was increased because the actual duration of Experiment 1 was longer than expected). All participants provided informed consent by clicking on a checkbox before starting the task.

#### Apparatus and stimuli

The stimuli were presented in an online task, using essentially the same experimental routines as in Experiment 1 (programmed in JavaScript using jsPsych).

A set of 20 young (age group 18–29 years), Caucasian faces was selected from a data base of adult facial stimuli (Minear & Park, 2004). Ten male and ten female faces were selected (according to the categorization provided by Minear & Park, 2004), all with neutral facial expressions. We opted for only Caucasian and only young faces to have a data set only characterized by one type of (widely inferred) group membership (gender). With these constraints, a fully random sample of facial images was drawn from the full set of 137 images in these categories (using the sample() function in R), thus avoiding any biases due to subjective selection by the experimenters.

#### Procedure

The procedure was identical to Experiment 1, except that only about half of the participants (*n* = 101) rated the 20 faces on a 21-point Likert scale before the pairwise comparisons (thus allowing us to compare unbiased pairwise comparisons with pairwise comparisons that followed direct evaluative ratings). All participants completed a full pairwise comparison of all 190 pairs of faces using the same procedures as in Experiment 1, and subsequently rated the faces on the Likert scale.

## Results

As in Experiment 1, the pairwise comparisons of faces were checked for (in)consistency in terms of circular triads (when *a* > *b*, *b* > *c*, but *a* < *c*) using the{eba} package for R (Wickelmaier, 2020; Wickelmaier & Schmid, 2004). On average, participants produced 53.2 circular triads out of a maximum of 330, but the number of circular triads was significantly below chance in all but one participant, who made *T* = 279 circular triads (Kendall’s *zeta* = 0.18; *p* = 0.34). As this indicated random choice behavior, the data of this participant were not included in the subsequent analyses. The cumulative preference matrix for the remaining participants is shown in Table 2, depicting the absolute numbers of preferences for each face in the rows compared to the respective faces in the columns of the table. Stochastic transitivity checks for all possible triads of stimuli in the cumulative preference matrix revealed only two violations of WST (in 1140 tests), which was not significantly different from the number of transitivity violations expected by chance alone, *D*(3) = 2.23; *p* = 0.53. Therefore, the pairwise preference judgments fulfill the assumptions to derive an ordinal scale representing the rank order of the liking of the faces. There were also very few violations of MST (6 triads; 0.5%) and SST (in 200 triads; 17.5%)—slightly less than in Experiment 1.

**Table 2:** Cumulative preference matrix (*N* = 196) for 20 faces, depicting the absolute numbers of participants who preferred the face in the row to the face in the column. Note that the original stimulus names were replaced by random alphanumeric labels with #F and #M referring to female and male faces, respectively

	#F56	#F13	#F25	#F62	#F82	#M36	#M75	#M54	#M72	#M29	#M84
#F56	0	90	37	64	98	84	148	125	109	96	104
#F13	106	0	40	60	95	100	157	137	113	99	113
#F25	159	156	0	103	149	145	170	160	155	139	151
#F62	132	136	93	0	126	133	167	157	144	130	143
#F82	98	101	47	70	0	90	148	131	104	100	104
#M36	112	96	51	63	106	0	159	147	110	92	123
#M75	48	39	26	29	48	37	0	52	49	37	44
#M54	71	59	36	39	65	49	144	0	69	73	74
#M72	87	83	41	52	92	86	147	127	0	91	104
#M29	100	97	57	66	96	104	159	123	105	0	127
#M84	92	83	45	53	92	73	152	122	92	69	0
#F24	165	173	123	135	158	152	180	173	165	149	171
#F85	156	162	100	107	149	136	171	167	162	135	154
#F14	113	108	34	64	110	111	152	129	115	99	124
#F35	164	174	133	137	166	158	182	181	172	154	174
#F20	125	128	55	79	112	112	155	141	124	103	127
#M77	41	33	14	21	26	29	98	49	33	42	24
#M33	51	42	26	27	41	35	104	76	54	55	55
#M60	76	73	28	41	67	62	127	97	74	69	84
#M93	37	35	17	21	30	26	91	46	31	34	34

	#F24	#F85	#F14	#F35	#F20	#M77	#M33	#M60	#M93
#F56	31	40	83	32	71	155	145	120	159
#F13	23	34	88	22	68	163	154	123	161
#F25	73	96	162	63	141	182	170	168	179
#F62	61	89	132	59	117	175	169	155	175
#F82	38	47	86	30	84	170	155	129	166
#M36	44	60	85	38	84	167	161	134	170
#M75	16	25	44	14	41	98	92	69	105
#M54	23	29	67	15	55	147	120	99	150
#M72	31	34	81	24	72	163	142	122	165
#M29	47	61	97	42	93	154	141	127	162
#M84	25	42	72	22	69	172	141	112	162
#F24	0	119	172	88	149	186	180	176	185
#F85	77	0	155	55	130	189	172	167	179
#F14	24	41	0	23	86	160	146	125	163
#F35	108	141	173	0	158	190	179	175	187
#F20	47	66	110	38	0	166	151	134	167
#M77	10	7	36	6	30	0	92	56	120
#M33	16	24	50	17	45	104	0	63	130
#M60	20	29	71	21	62	140	133	0	155
#M93	11	17	33	9	29	76	66	41	0

The BTL model was again fitted to the preference probabilities to derive *u*-scale values of the 20 face likings. However, in contrast to Experiment 1, the maximum-likelihood test revealed that the face preference judgments deviated significantly from the restrictive BTL model, *G*^2^(171) = 225.3; *p* = 0.003, indicating that the BTL model does not provide an accurate description of the data. Therefore, a less restrictive EBA model was fitted to the data, including the gender of the faces as an additional aspect in the model structure. The maximum-likelihood test revealed that the data did not deviate significantly from this model, *G*^2^(170) = 196.9; *p* = 0.08, and a model comparison confirmed that this EBA model (AIC = 1253) provided a more accurate description of the data than the BTL model (AIC = 1279), *D*(170) = 28.46; *p* < 0.001.

**Fig. 4:**
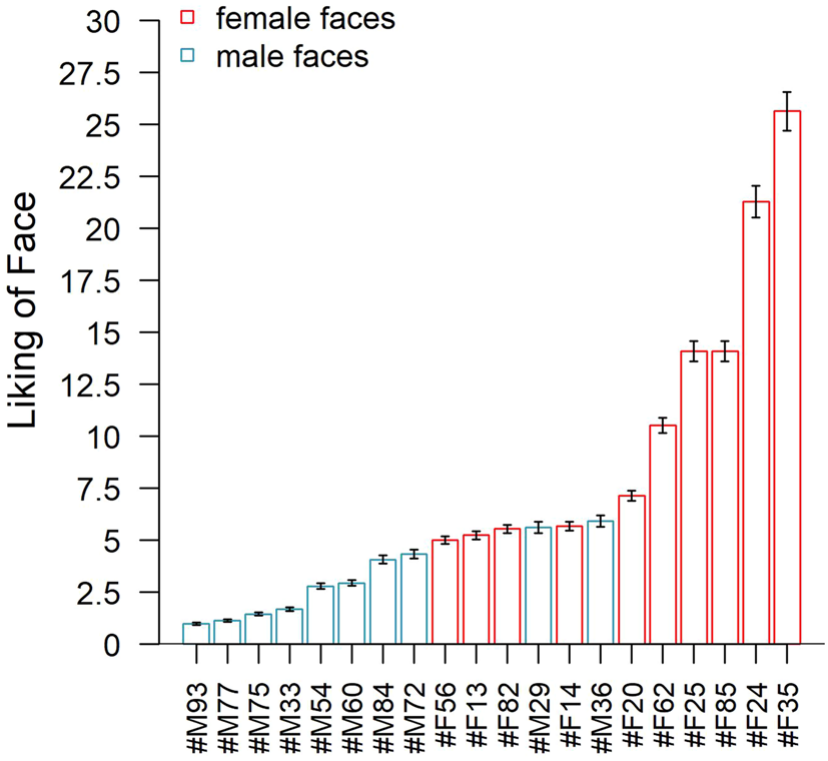
Estimated ratio-scale values for the subjective liking of male and female faces based on an elimination-by-aspects (EBA) model with the gender of face as the only aspect in addition to the identity of faces. Error bars indicate standard errors of the model estimates. Names of the facial stimuli were replaced by random alphanumeric labels, with #F and #M referring to female and male faces, respectively

Based on the EBA model, *u*-scale values were estimated for the subjective liking of all 20 faces and normalized with regard to the face with the lowest liking, which was assigned a value of 1 (the reference). The resulting *u*-values of the liking of the 20 faces are illustrated in Fig. 4, revealing a considerable range of likings. Specifically, the most liked face (#F35) was found to be liked 25.6 times more than the reference face (#M93).

**Fig. 5:**
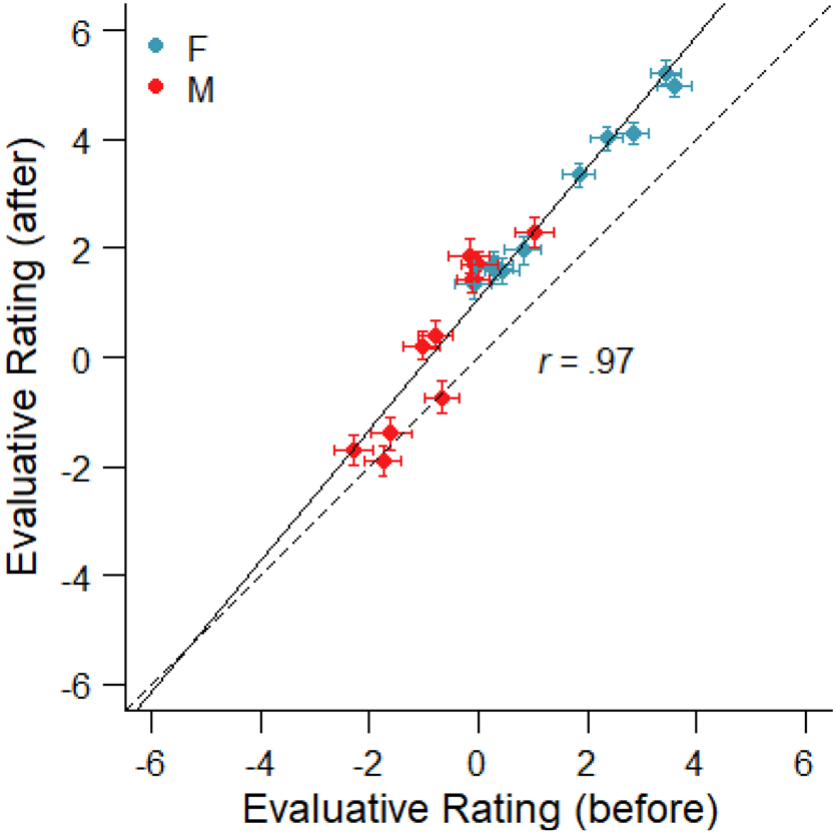
Correlation of evaluative ratings on a Likert scale from − 10 to + 10 given before and after the pairwise preference judgments for the 20 facial stimuli presented in Experiment 2. Error bars indicate standard errors of the means. The solid line represents the linear regression, and the dashed line represents the diagonal

The average evaluative ratings in the first rating phase ranged between *M* = − 2.29 (SD = 3.52) and *M* = 3.60 (SD = 3.09). In the 100 participants who rated the faces only after the pairwise comparisons, the average evaluative ratings ranged between *M* = − 2.05 (SD = 3.95) and *M* = 5.56 (SD = 2.88). As in Experiment 1, there was a strong and significant correlation between the first and second evaluative ratings (in participants who rated the faces both before and after the pairwise comparisons), *r* = 0.97; *t*(18) = 18.14; *p* < 0.001, indicating high re-test reliability for the evaluative ratings of faces. In addition, a 2 (time of measurement) × 2 (face gender) repeated-measures ANOVA on evaluative ratings revealed a significant increase from the first (*M* = − 0.38; SD = 1.72) to the second ratings (*M* = 1.47; SD = 1.75), *F*(1,95) = 52.22; *p* < 0.001; *η*^2^_G_ = 0.06, (compare Fig. 5). The evaluative ratings also differed significantly between male (*M* = -0.29; SD = 1.99) and female faces (*M* = 2.14; SD = 1.86), *F*(1,95) = 115.97; *p* < 0.001; *η*^2^_G_ = 0.25. However, there was no interaction between time of measurement and the gender of the face, *F*(1,95) = 0.16; *p* = 0.69; *η*^2^_G_ < 0.01, indicating that the face gender difference did not change with repeated evaluative ratings (in contrast to the differences between artists observed in Experiment 1).

**Fig. 6:**
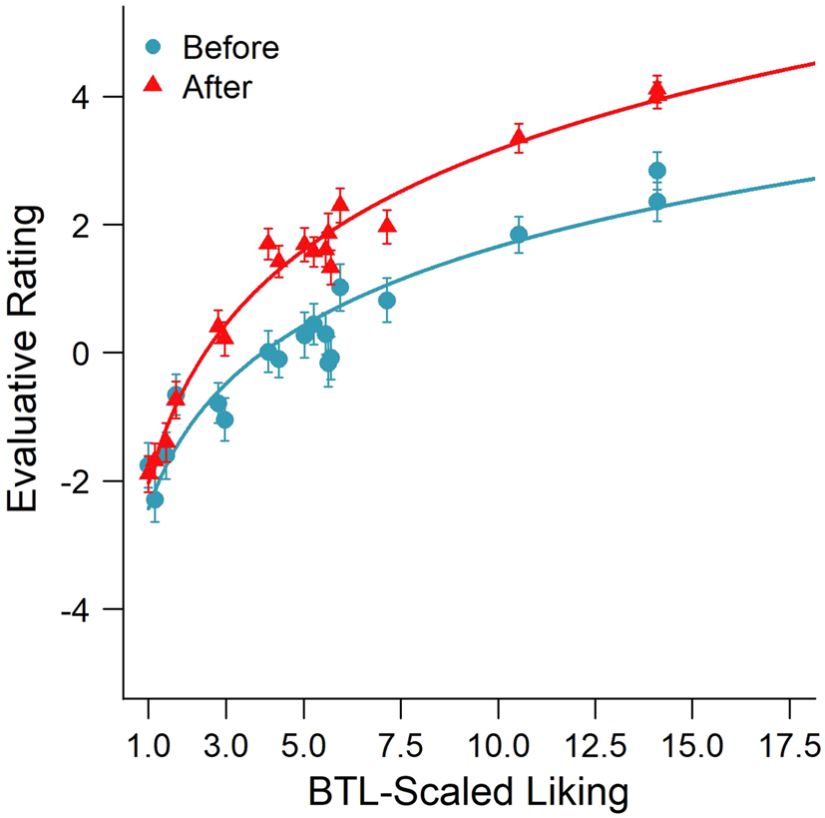
Evaluative ratings of faces before and after the pairwise comparisons as a function of ratio-scaled liking weights that was estimated by an elimination-by-aspects (EBA) model of pairwise preference judgments. The solid line is the best-fitting logarithmic regression predicting the first and second ratings as a function of the liking scores based on the EBA model

Figure 6 shows the direct evaluative rating before and after the pairwise preference judgments (including only the ‘after’ ratings of participants, who did not rate the faces before) as a function of the *u*-scale values estimated by the EBA model. As in Experiment 1, the evaluative ratings could be predicted quite accurately as a logarithmic function of the *u-*scale values of liking, both for the ratings before (*a* = 4.08; *b* = 3.93; *p* < 0.001) and after the pairwise comparisons (*a* = 5.70; *b* = 2.48; *p* < 0.001). A linear regression of the evaluative ratings as a function of the logarithm of the *u*-values also provided a very good fit of the data, accounting for about 93% of the variance for the ratings that were given before (*R*^2^ = 0.93) and 98% of the variance for the ratings after the pairwise comparisons (*R*^2^ = 0.98).

Based on such a strong relationship between the evaluative ratings on the Likert scale and the underlying liking weights of the stimuli (as derived with the EBA model), it might be possible also to derive a generalizable function to estimate the liking on a ratio scale based on the evaluative ratings alone, without having to complete a full pairwise comparison. In the present example, the *u*-scale values of liking can be predicted reliably as an exponential function of the first and second ratings (see Eqs. 7 and 8), accounting for 89% and 92% of the variance in direct evaluative ratings.

7 $$u=3.76+0.60\cdot {e}^{ER1},$$

8 $$u=3.87+0.09\cdot {e}^{ER2}.$$

**Fig. 7:**
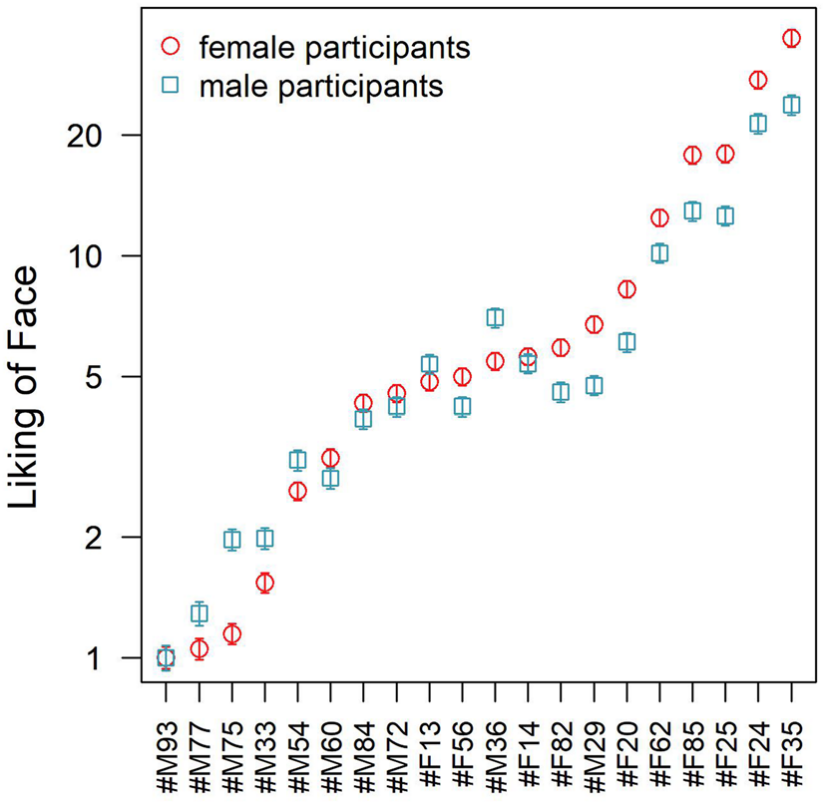
Estimated ratio-scale values (log scale) for the subjective liking of faces based separate restrictive BTL models fitted to male and female participants (ordered with regard to the female participants’ liking scores). Error bars indicate standard errors of the model estimates. Note that #M and #F in the labels on the abscissa refers to male and female faces, respectively

As face gender turned out to be a crucial aspect for preference judgments, separate probabilistic choice models were fitted also for female and male participants. Interestingly, the likelihood ratio test revealed that in these separate analyses the restrictive BTL model (i.e., a probabilistic choice model without an aspect for the gender of the faces) provided a very good fit to the face preferences in both female *G*^2^(171) = 160.8; *p* = 0.70, and male participants, *G*^2^(171) = 160.6; *p* = 0.70. The *u*-scale values estimated with the two separate models are illustrated in Fig. 7 (note the log scale), indicating that male and female participants’ liking of facial stimuli exhibited slightly different rank orders. While there was no general difference in *u*-values between male and female participants across all face stimuli, *t*(38) = 0.66; *p* = 0.51, it appears that women had more extreme attitudes towards the most “liked” and most “disliked” faces compared to men, whereas the gender differences were less systematic for the faces in the mid-range (see Fig. 7).

## Discussion

Experiment 2 showed that the highly restrictive BTL model failed to derive a valid ratio-scale representation of the subjective liking of facial stimuli from the preferences judgments in male and female participants together, suggesting that facial photographs of young Caucasian women and men (in contrast to the paintings from different artists used in Experiment 1) are evaluated based on multiple discriminating aspects, which are difficult to integrate on a unidimensional scale. For the present set of male and female faces, it is plausible that such a discriminating aspect might be the gender of the face, and we found that an EBA model accounting for gender as an additional aspect provided a more accurate description of the data (note that this cannot be done in the more restrictive BTL model). Hence, it was possible to derive a valid ratio-scale representation of the liking of facial pictures, and these liking scores could be related to the corresponding direct evaluative ratings. In addition, two separate BTL models fitted separately to male and female participants’ preference probabilities revealed an even better description of the preference judgment data.

Consistent with Experiment 1, it was found that direct evaluative ratings can be described quite accurately as a logarithmic function of the indirectly obtained liking scores on a ratio scale (accounting for more than 90% of the variance in evaluative ratings given either before or after the pairwise comparisons). This suggests again that differences and ratios of numerical values on the evaluative rating scale must not be interpreted at face value, but an exponential transformation can be used to derive liking scores that are valid a ratio scale (as the *u*-values derived through probabilistic choice models).

## General discussion

Two experiments demonstrated that a ratio-scale representation of the subjective liking of stimuli can be derived from the preference judgments in full pairwise comparisons based on a probabilistic choice model. In Experiment 1, the liking of paintings from four different artists could be scaled with the highly restrictive BTL model, revealing a factor 16 between the liking of most disliked and the most liked painting. In Experiment 2, we found that the liking of male and female faces could be scaled on a ratio scale either with an EBA model accounting for the gender of the face as an additional aspect, or with two different BTL models that were fitted separately to the judgments of male and female participants.

In general, these findings indicate that affective preferences can be judged consistently for a given set of stimuli, both across participants and across multiple binary comparisons, allowing the derivation of a ratio-scale representation of the subjective liking weights of the stimuli. In contrast to a scale obtained from direct evaluative ratings, this scale of liking scores is based on the statistical assumptions of a probabilistic choice model, which had been tested for two independent data sets—and there is always a chance for the model to fail. In fact, while the restrictive BTL model provided a good fit to the preference judgments for abstract paintings in Experiment 1, it failed for the preference judgments of facial stimuli in Experiment 2. This suggests that pictures of human faces may be a stimulus set that is too heterogeneous and characterized by multiple attributes (aspects), which may lead to the formation of subgroups of similar stimuli (e.g., male and female faces) and possibly prevents the integration of different features or aspects on a unidimenstional liking scale. In the resulting multidimensional stimulus space, violations of the assumption of context independence become more likely with an increasing number and variety of to-be-compared stimuli (Tversky & Sattath, 1979). The finding that even a relatively homogenous stimulus set (young Caucasian faces) with only one clear categorical division (gender) can lead to inconsistent preference judgments has implications for research areas where stimulus sets that are structured into subgroups are to be evaluated on the same scale (e.g., investigating prejudice against groups or temporally unstable preferences for healthy or unhealthy food). However, even a slightly more complex EBA model with a single additional aspect for the gender of the face could be fitted successfully to the preference data, indicating that the judgments were highly consistent despite the existence of a multidimensional stimulus space.

In addition to the mathematical foundation of scaling, the degree of liking of the stimuli on the resulting scale can be interpreted in terms of mathematical ratios, allowing statements such as “painting A is liked eight times better than painting B”. In contrast, such an interpretation is not valid for the numerical liking scores obtained through direct evaluative ratings. However, in the present study, the evaluative ratings were related to the liking scale based on the probabilistic choice models, indicating that the evaluative ratings can be described as a logarithmic function of the underlying ratio-scaled liking score. Hence, the multiplication of a liking with a particular factor (e.g., to like a stimulus twice as much) will result in a constant increment on the evaluative rating scale. Such a non-linear relationship between an underlying sensation and direct reports on a numerical scale (e.g., magnitude estimation) has been observed in many psychophysical studies (Stevens & Galanter, 1957), and more recently also for affective dimensions (Zimmer et al., 2004).

## Open practices statement

The data of the present two experiments (pairwise comparisons and direct evaluative ratings of paintings and faces, contained in csv-files) and the analysis code (R) are openly available as a working example in an Open Science Framework (OSF) repository at this link: https://osf.io/vt6gb/?view_only=846ecab0215e453dad08dfe961d848a5

The analysis code can be used to fit two types of probabilistic choice model to preference judgments (using the {eba} package): the BTL model and an EBA model with one additional aspect. In addition, the code involves frequentist statistics (e.g., using the {ez} package), and allows to reproduce the main figures from this article. Additional information such as the experimental routines (JsPsych) can be made available upon request.

## Footnotes

1 We use ‘subjective liking’ as a term for the evaluative tendency or representation that a participant holds for a stimulus and which is then translated into an evaluative response. We decided not to use the more common term ‘attitude’ as it sometimes contains non-evaluative aspects as well.
